# Observations on the Topography, Climate, and Diseases of a District in the Eastern Part of the State of Ohio

**Published:** 1842-01

**Authors:** Thomas Carroll

**Affiliations:** Late of St. Clairsville, now of Cincinnati, Ohio


Art. III.—*Observations on the Topography, Climate, and Diseases of a District in the Eastern part of the State of Ohio.* By Thomas Carroll, M. D., late of St. Clairsville, now of Cincinnati, Ohio.

This district of country lies between 39° 25', and 40° 46' north lat. Its mean breadth is about fifty miles. It is the most elevated part of the State of Ohio; the highest points being not less than one hundred and twenty feet higher than those of any other section of the State. The dividing ridge running through this tract of country approaches much nearer the Ohio than it does the rivers on the west of it. Those streams falling into the Ohio have, of course, much more velocity than those that run into the Muskingum and Tuscarawas. Several of these tributaries have a retrograde direction, which renders their velocity still less; notwithstanding the slowness of their currents, they have cut deeply into the strata, leaving high elevations on either side. Their alluviums are often extensive and subject to inundation at various seasons; this liability is much increased in consequence of the elevation and great unevenness of the country. The alluviums along the Ohio and its tributaries are not very extensive and are but seldom inundated.

With the exception of a small tract, south of Sandy creek, every portion of this district is hilly. The altitude at which most of the population live above the level of the ocean is from eleven to twelve hundred feet; being about five hundred above the Ohio river, whilst some are six or seven, perhaps, even more. But a small proportion of the population reside on the alluviums. The elevation of the country renders it but little subject to fogs; they are, however, frequent along the rivers and larger streams, but do not often mount more than half way up the hills. Above the elevation to which they reach, the air is dry and pure; hurricanes are not frequent, and when they do exist, they are not so violent as in the lower and more level parts of the State. The thermometer seldom or never rises so high nor falls so low as it does farther west. The position of the district secures it from the bleak winds that sweep over the lakes; and its elevation seems to guard it from those long continued droughts to which, I think, the less elevated and level parts of the west are subject; I, however, have but few data to rest this opinion on. Of the natives of this district it may be said (probably with truth) that they are the most beautiful of the natives northwest of the Ohio river. They are tall, muscular, well proportioned, and evince much intellectual power.

In common with Western Pennsylvania, and some other elevated sections of country the inhabitants of this district are subject to goitre. Its prevalence, however, is much less than it was thirty years ago; and when it does occur it does not often reach the same magnitude that it did formerly. As it has declined, pulmonary consumption has increased. This fatal affection was once of rare occurrence, but of late years many fall victims to it. The exchange of goitre for this disease is of course unfavourable to the interests of human life.

My friend, Professor Darby, of Canonsburgh, Pa., informs me that he had examined the burying grounds of different districts of country for the purpose of discovering at what periods of life the greatest amount of deaths had taken place, and he found that, in Western Pennsylvania, a larger proportion, from fifteen to twenty, had occurred than he had observed any where else. This mortality was, no doubt, brought about, in a great measure, by pulmonary affections; indeed it must be conceded that the inhabitants of countries located like those under consideration have always been subject to pulmonary affections.

Eastern Ohio, in common with the west, has undergone a change within the last forty years. When the forests were unbroken but little more than the gentle breeze was felt, but as the lands were cleared off more rapid currents of air became common. Greater vicissitudes of heat and cold took place, and they increase as improvement advances, which must, of course, have an influence on the human constitution. But the people have materially changed their mode of living, their manner of dress, and above all their education has undergone an immense change. Formerly females received but a slight education. A few months, during a very few winters, was all the time the girl spent at school, and frequently this was only continued for a single winter; and not unfrequently indeed her education was altogether denied. This condition of things slowly gave way, and that chiefly among the wealthier classes, to what is called a refined education. A young lady is now instructed in the country schools to read and write, with a slight knowledge of arithmetic; she is then sent to a boarding school for the purpose of having her education finished. Here she is required to spend a very large amount of her time in study, and the little exercise she takes is under peculiar restraints, which detract much from its beneficial influence. Had the pupil to endure this mode of life for a few months only she would most likely escape the awful consequences which follow, but unfortunately this course is pursued for years; one of the evils most to be lamented is, that this boarding-school part of her education is commenced at a most unfortunate period, (about the age of twelve or fourteen) and is often continued for two, three, or four years, through a period of life in which, above all others the utmost attention should be paid to the health of females. This unfortunate period, chosen for the education of girls, and the still more unfortunate manner, pursued in disposing of their time, lays the foundation of most serious mischief to their constitutions, and seems to have a peculiar tendency to derange the healthy condition of the lungs. When a *poor girl* becomes diseased, during the time she is finishing her education from home, and when her parents and teachers feel ambitious that her mental improvement should continue, but little attention is paid to her health, either by herself or her teachers. Paleness, loss of flesh and debility, do not give alarm; but at length a cough supervenes, or spitting of blood; when, for the first time, the fears of her friends are awakened, and she is sent home to receive the last charities of life. It must, however, be admitted that many others fall victims to diseases of the lungs; this is peculiarly the case with those who have been long confined to a sedentary life. Females, I think, often bring on pulmonary complaints, by long confinement to the house; and I am not certain but that the habit, which so much prevails of late years, of excluding solar light from our houses, has not had a tendency to increase these affections, and some other forms of disease.

The changes in the diseases of the male sex, in the region of which I speak, have probably not been so great as in those of the female, and are somewhat of a different kind. During the early settlement of the south-eastern part of Ohio, suffering and labor were endured which are now but seldom experienced. Forty years ago, few of the arts of civilized life were practised west of the Alleghany mountains. Every foreign fabric was dear, and rendered doubly so, in consequence of the scarcity of money; they were, therefore, nearly excluded from the country. Shoes were but seldom worn by males under the age of fifteen, except in winter, and often not by females; hence the liability to inflammatory disease at that time. Rheumatism was among the most prevalent of this class, indeed it was so common that the sufferer but seldom had much done for his relief, and was not unfrequently laughed at when he complained. As the improvements of the country advanced, more care was taken as to the clothing of children; houses became better, and labour less severe; of course this disease measurably subsided among the young, but many, even now, who have borne the burden of settling the country, when it was a wilderness, are severely afflicted with the disease in its chronic form. But few of the young men now lead a sedentary life, nor have they at any time, unless their residence was in towns, or they were designed for one of the learned professions; such have, sometimes, become victims of pulmonary disease, or have been subjected to dyspepsia, &c. In those who devote themselves to study, these diseases are almost incurable, disinclination to exercise, and an inclination to the use of tobacco, seeming to me to be among the first causes of them.

A diseased state of the ligaments and cartilages of the joints is probably about as prevalent here as in other sections of the Union, out of large towns and cities, from which the female suffers about as much as the male. I am inclined to the opinion, that the osseous structure, particularly of the vertebra, has suffered to a greater extent among the people of eastern Ohio, during the past forty years than in other parts where less severe labour has heen endured, as I have known several instances of diseased spine which manifestly had their origin from severe lifting.

During the colder season of the year a singular species of inflammatory disease is occasionally met with, and sometimes frequently. It consists of an inflammatory affection of one side of the head, attended with fever; the blood, when drawn, exhibiting a cupped and buffy coat. The eye and ear of the side affected are most pained. There are throbbing and intolarence of light, accompanied with fulness or swelling of the side of the head. The pain is more intense during the after part of the day and fore part of the night; subsiding towards morning, and again increasing towards noon. Sometimes this disease, or one similar to it, is met with in nervous females during the spring and summer, and when it appears in them it seems to assume the character incident to a similar affection in districts, subject to bilious diseases and may sometimes be relieved by the same treatment, but the inflammatory kind must be managed in a different way. As the complaint will generally continue for at least ten days it will be necessary to take this into consideration when the treatment shall be commenced. The reason why this affection continues so long, is, that the fluids have to undergo, as in other inflammatory diseases, a radical change before health can be completely restored.

In the treatment bleeding should first be had recourse to; then purging, followed by antimonials, which should be given throughout the complaint, in doses so moderate that much sickness will not be produced, if any. If the pain in the eye or ear be very great, taking blood from the temporal artery will be of great service; cupping, after the system has lost enough by general bleeding, will be found very serviceable. It should be recollected that this disease is attended with severe inflammatory symptoms, and that there is always danger of inflammation of the brain, or its membranes, unless an active treatment be adopted.

The remitting and intermitting fevers of this region were, during the early settlement of the country, regular summer and autumnal visiters of the valleys of the Ohio, Muskingum, Tuscarawas, and of those of most of their large tributaries. With the advance of improvement, the disposition to these fevers has lessened until it has almost disappeared along the Ohio and its small tributaries. This, however, is not the case on the alluviums of the other streams. It will probably be long before these latter localities can claim an exemption from them. The total disappearance of drift-wood from these waters, and the straightening and embanking of some of them, would, in all probability, render their margins healthy to a much greater extent, than they now are; as inundation would by these means be in a great measure prevented. Many years must, however, elapse before that attention will be paid to this matter which will insure a general exemption from these fevers.

The inhabitants of the high lands, are very seldom affected with these diseases, particularly with intermitting fever; unless they expose themselves to the miasma of low situations. The high grounds, however, lying to the northeast of extensive low lands sometimes subject their inhabitants to these fevers. I recollect a locality very near New Lisbon, in Columbiana county, where persons were attacked at the distance of a mile and a half from the origin of the malaria, notwithstanding that a dense forest intervened. The origin of the malaria was, however, to be found in the extensive flooding by a mill-dam of a wooded alluvium. Take the country for twenty miles west of the Ohio, and extending from the Pennsylvania line to Marietta, and, I apprehend, no district, between it and the Mississippi has so great an exemption from these fevers. Indeed, no country could be much better calculated to restore to perfect health constitutions partially broken down by remitting and intermitting fevers.

Autumnal fevers, when they occur, have more of the continued than of the remitting type; and seem to run insensibly into the continued fevers which appear late in the fall, and during the winter. Of these fevers I shall speak by and by, but shall now proceed to make a few observations on dysentery, which occasionally visits this region during the summer and autumn; and is not unfrequently severe and fatal. Dysentery most usually makes its appearance during dry weather, when the smaller streams are nearly dried up. I have not been able to discover, that this disease is owing to improper indulgence in summer or autumnal fruits, or in any particular diet, though it is no doubt occasionally *excited* by improper food. I have, several times, known it to occur in neighborhoods where fruit was rare and where the inhabitants lived on simple food. I think it is more frequently rife when vegetation is rendered languid by long continued drought, or when it begins to decline during autumn. Dysentery seems here to be governed by laws very nearly connected with those that govern autumnal fevers; this is particularly the case with regard to its duration.

The dysentery of this section has more intensity of character than it has in miasmatic localities. The fever is more continuous and the pain more constant; hence there is more fatality in the same number of cases. In its acute form but few diseases proceed with more rapidity to a fatal termination, and very few cause so much suffering. The subacute form is more tolerable as the patient has occasional intermissions of pain. In a few of the worst cases of acute dysentery, which I have seen, the patient had no respite from pain till the approach of death. In confirmation of this, I shall give the following case:

Miss G., aged 23, was taken, in September, 1829, with dysentery. The pain was unremitting from the first, and the evacuations frequent and bloody, accompanied with tenesmus, and great tenderness of the abdomen. In consequence of the condition of the abdominal viscera, the breathing was short and difficult; there was sweating of the face, with moisture of the surface generally, which was about the natural temperature. I saw this unfortunate patient on the third day of her illness, for the first time. She was bled and purged; calomel and opium were administered in large doses, accompanied with cupping and the warm bath, but all without effect. She died thirty-six hours after I first saw her. But little mitigation of the pain was produced by any medicine or prescription that was made.

Children more frequently die of this disease than adults, and observation has led me to conclude that females fall victims to it more frequently than males. But few adults, however, are lost when the treatment is early commenced and persued judiciously throughout. This is peculiarly the case in the treatment of the subacute form.

Humanity owes much to the science of medicine for the improved treatment of this disease, yet old methods of cure still have their place and that which is inert or absolutely injurious is sometimes prescribed with all the solemnity attendant on the exhibition of the most potent and appropriate medicines.

Among the prescriptions that are compartively inert, may be counted the various mucilaginous drinks, such as those from gum arabic, and slippery-elm bark. A common prescription in the country, even now, is mutton soup and other articles of a similar kind which are administered whenever the sufferer can receive them without vomiting; and those are given from the very onset of the disease. I think it must be admitted that physicians are too careless about matters of this kind, for they sometimes approve of small quantities and often suffer their patients to be fed to death without saying a word against it. Can they believe that murder, committed by stuffing the stomach when the colon is inflamed, is not a crime?

I would gladly pass over without reprobation another prescription, which physicians cling to with much greater tenacity than their patients—I mean injections. I am not certain that *I* ought to judge of them, as I must confess that I long since became prejudiced against their use by their effects in my own case. I never look back to the period when I was tortured by them without having a worse feeling against them than any man ought to have against an enemy. Opiates given by the mouth seem to me always to have as soothing a tendency as when administered by injection; and then all the horrible irritation which follows injections is avoided.

From the description I have given of dysentery, as it occasionally has occurred in this part of the country, it will no doubt be admitted that, under similar circumstances, an active treatment should be adopted and persevered in for some days, particularly in the acute forms of the disease. The course which I usually adopted in severe cases was to bleed freely at the onset, then to give an emetic of tartarized antimony, or ipecacuanha and calomel. As soon as vomiting had ceased a cathartic was administered of calomel followed by sulphate of magnesia; or jalap in strong constitutions, was substituted for the salts. When the cathartic had operated about twice pretty freely, calomel and opium were given in the proportion of ten grains of the former and one of the latter, with half a grain of ipecacuanha. About double this amount was administered at once, and repeated every four, six, or eight hours, as necessity required, until the mouth became affected with the mercury, or other specific effects were produced. It was often necessary to watch, with care, the effects that this medicine produced; for salivation was not always produced even by the largest doses, as many patients were not susceptible of its salivating operation. Such patients were, however, susceptible, very frequently of the constitutional operation of mercury, which could be known by the general condition of the system. In subacute cases smaller quantities of both opium and calomel were found sufficient; for instance, five grains of calomel, with one of opium, and half a grain of ipecacuanha, was observed to be enough for patients whose constitution, had been broken down by using mercury to excess. Sometimes the blue pill was substituted, in ten grain doses, with Dover's powder, or what was better, sulphate of morphia and ipecac., which were found to answer better with nervous subjects. During the time this treatment was used attention was also paid to the skin. The warm bath was used twice a day, or oftener when it was practicable, and was found of much service. When the bath could not be used fomentations were substituted and were found of much service, indeed they were applied whether general bathing could be employed or not. But few general bleedings can be useful in most cases of dysentery, and so soon as the lancet is laid aside, local bleeding, by cups, is important, and I often found this method of drawing blood of great use. If there was much blood discharged and great soreness of the abdomen, I mostly found cupping useful; where great debility is present it will be of much service to use dry cupping; about half a dozen cups, set over the abdomen, will invite considerable blood to the surface, and I have often found that much relief from soreness and pain was experienced by the patient. It will probably be unimportant for me to say anything about the treatment that was pursued with most advantage in the close of the disease, when mucus and blood were no longer discharged, and where a diarrhœa had set in. I may remark, however, that acetate of lead with cretaceous preparations and opiates were then most to be relied on.

In long continued cases I have often found blistering of service, and sometimes warm brandy and laudanum have a happy effect when used as a fomentation over the abdomen during the night.

The reader has above observed the recommendation of epsom salts as a cathartic in dysentery; this recommendation grows out of long experience in the use of this medicine. I thought, many years ago, that hydragogue cathartics had more influence, when judiciously combined with mercurials, in overcoming the morbid action of the bowels than any other cathartics, and more extended experience still has confirmed me in my former opinion.

The continued fevers of this region are more to be feared than any others, unless it be the eruptive; and probably no change of living nor alteration of the face of the country will ever eradicate them. Their existence is not determined by the season of the year, however they mostly commence in the latter part of autumn, when their characteristics approach more to those of remitting fevers than they do at other seasons. When they commence in autum, they often continue into the winter, or even through that season; and when they begin in winter, they frequently run on till spring. I recollect that one of these fevers began in the fall or early part of winter and continued through the ensuing spring and summer. This was in the year 1814, before I studied medicine, so that I can say but little about the true nature of it. I believe, however, that it wore a different aspect, in some particulars, from the winter fevers that have since appeared.

These continued fevers seem to depend upon a different cause or causes, from those which give origin to remitting and intermitting fevers; the former are found mostly on the high lands, and more often on those that are sterile than the rich; whilst the latter infest the alluvial districts, or marshy plains. In the common acceptation of the word, most of this district is infertile; the lands having a clay substratum often combined with a smaller or greater proportion of iron, but with little or no lime in their composition. Portions of the country, it is true, are rich, but they are not often of great extent; yet, probably one third of the *whole* may be considered good and some of it very productive. Lands of the latter description are clothed, when uncultivated, with native forests of great luxuriance and beauty, composed of walnut, sugar maple, buckeye, and other trees which delight in a fertile soil. These rich soils have mostly a considerable quantity of lime in their composition. The springs here have more lime in their waters than exists in those of the poorer sections; and they are generally more abundant.

As a medical man I observed the continued fever, for the first time, in November, 1823. It then existed near New Lisbon, and on the high and poor lands south of that town. In this locality it was peculiarly mortal. I again witnessed it at St. Clairsville, Belmont county, and the immediate neighborhood, late in the autumn of 1826. Here the land is of the finest quality, and the water limestone. I attended more than fifty cases within a very small space. It has, within the last sixteen years, frequently appeared in different portions of the southern and western parts of Belmont county, and also in Jefferson, Harrison, and other contiguous counties, no doubt oftener during winter than in any other season.

This fever often begins in a single individual of a family, and no other instance will be met with for a week or two, when one or more of the same family will be taken ill, and after some days, one or more of those who visit and attend on the sick, will, in their turn, sicken, and their friends, after attending them for some days, will also be taken down. In this way this fever seems to propagate itself. No people, probably, pay more attention to the sick than the members of the *Society* of *Friends*, and among them I have noticed the winter fevers to be peculiarly frequent; and have observed it extended to a great number. These facts have had a strong tendency to make me believe that this fever could be communicated from one individual to another. Further observation will, no doubt, decide this matter. The temperate, as well as the intemperate, are subject to its ravages. Circumstances in the mode of living seem to make but little difference, and children, as well as adults are obnoxious to it.

When an individual is about being taken with this fever he appears languid, has a bad appetite, and complains sometimes of a headache, and again of pain in the back; inclines to sleep, though his slumbers are disturbed, and he rises from them without feeling refreshed. After some days spent in this way, he gets out of bed but seldom, eats little or none, and his mind sometimes wanders when he is aroused. In a day or two more he lies all the time unless his brain be seriously affected, when he is often much inclined to sit up, even when very ill. If his skin be examined during this time it will be found dry and inactive, and when pinched into folds it regains its smoothness very slowly, showing the loss of its elasticity. There is commonly but little flushing of the face at the commencement of the disease, but, as the fever advances, this symptom becomes more developed, especially in the after part of the day, and in some cases is eventually relieved by sweating. If the brain be diseased early, he will often show slight delirium, and will mostly complain of disagreeable feelings when he shakes his head, which he will not do without being urged; the eyes frequently become more brilliant than usual, and there is sometimes morbid acuteness of vision, with suffusion of the conjunctiva. Headache is not always present when the brain or its membranes are affected, and it is often present when the result shows that these parts were very little, if at all, inflamed. When the patient gets down it will be found that the fever is regularly augmented in the afternoon, and during the fore part of the night, when it begins to become more mild and abates considerably against morning; but is again renewed with the same result as before. It, however, is commonly more severe, every other day throughout the course. During the continuance of the fever, the surface is *generally* somewhat above the natural temperature, though it is often not warmer than in health, and frequently as cool or even cooler. Sometimes this fever appears in an acute form, running its course in a few days. In these cases, the brain or heart is most deeply affected, sometimes one of these organs being more diseased and sometimes the other. When the heart is affected, the pulse has an irregular beat, and seems corded, and occasionally intermits; but when the brain is affected there are delirium, pain in the head, with subsultus, intolerence of light, &c. The acute form is sometimes occasionally ushered in with chills. This symptom is, however, seldom observed at the commencement of the subacute. A dry tongue is very common at all periods, but especially in the latter, when it is often brown in the centre, with a flabby inactive appearance. When the patient attempts to put it out it seems to catch on the teeth. It is sometimes pointed. The former of these symptoms is unfavorable, the latter more so.

It is, probably, unnecessary to say any thing now about the symptoms which mark a favorable termination of the fever. I will, however, make a few remarks on those that are unfavorable. Great irritability of the stomach with pain in it and the bowels, and tenderness of the epigastrium and abdomen generally, are unfavorable; hemorrhage from the bowels is among the most unfavorable symptoms; and still more so is a swelling of the penis; indeed I have but seldom seen a recovery from fever when this symptom supervenes. The want of the tonic contraction of the cremaster muscles is also unfavorable; when this is the case, the testes are found low in the scrotum which is also relaxed. I am inclined to think that when these two last symptoms are present the brain is incurably affected. It might be improper for me to pass over another symptom that I have often witnessed. When you lift one of the forearms, so that it forms with the arm a right angle, and hold it there a few moments, and then take your hand away, if it be retained by the patient in this position, voluntarily, until it begins to tremble, and then falls without a voluntary act of the patient, it may be considered that danger is present and the chances of recovery are few.

We will now take a view of the treatment that should be pursued in the cure of this fever. It must always be borne in mind that it has a duration of from seven days to seven weeks, and is sometimes extended much farther, for, by leaving this matter out of the question, I think I have seen no little damage done. If the physician begins the treatment with an idea that he will terminate the fever in a few days by medicine he will be almost certain to do injury, and will often have to submit to the mortification of losing his patient. It is a matter then of moment that, when the treatment is begun, the physician should come to the conclusion at the outset that he will only attempt to moderate the force of the fever, and that he will direct such means as will have a tendency to establish a healthy condition of the secretions and excretions by degrees; always recollecting that local irritations should be relieved by local as well as general means. By taking a course of this kind very few patients will be lost, because the physician sees a-head and wards off the dangers that lie in the way, whilst he knows that if he ever exhausts the powers of the system it must sink before the disease arrives at the goal pointed out by nature. I would not be willing to say that this fever is in no case arrested in its origin by a decided course of treatment, but I well know how much disappointment I have witnessed growing out of such a course.

The first step to be taken in the acute form of this fever is to bleed until the patient either approaches to syncope or does faint; to effect this, without danger, he should be in a sitting or erect position when bled. As soon as he partially recovers from the effect of the loss of blood, he should be placed in a sitting position, when two or three gallons of cold water ought to be dashed over him; if his head be much affected it should be allowed to fall in a heavy stream on it, for by this means it will be much relieved. It then becomes necessary to administer either a cathartic or an emetic. If the patient have a foul tongue and his head be not much affected, the latter should be preferred; but if these circumstances do not exist the former should be chosen. Should the physician determine in favor of an emetic he will consult the interests of his patient by giving him, in some warm water, half a grain of tartar emetic, and one or two grains of ipecacuanha, combined, every half hour until vomiting is effected. It is now important that a powerful cathartic should be given, composed, in most cases, of calomel and jalap, or for the jalap, castor oil or senna and salts, or, in weak constitutions, rhubarb. After a free cathartic effect has been obtained, tartar emetic should be given in such doses as will not sicken, and be persevered in throughout the disease, unless it should be found that it does not agree with the patient. But few cases will be met with, however, where a twelfth of a grain cannot be borne, and this amount will be sufficient to begin with, and should it be borne with ease, the dose can be increased as occasion may require. It should be given every four or six hours. After a free cathartic influence has been obtained, twenty grains of calomel should also be given and repeated every six or eight hours, until the mouth becomes sore, or the specific effect is produced on the general system; for many cases will be met with where the mouth cannot be made sore, yet the constitutional influence of the mercury will be obtained. When the calomel has been given until it has produced its specific effect, no more should afterwards be exhibited than enough to keep the secretions of the alimentary canal right. Two or three grains a day will be found sufficient for this purpose, and if profuse salivation should occur, no more should be given for at least a week. It has been a favorite opinion with many, that when salivation is once produced, the fever of course ceases. This opinion is, however, fallacious, for nothing is more common than to see the fever go on for weeks after the specific influence of mercury has been induced. It is true that it is generally mitigated by the mercurial action, but by no means always destroyed.

When the disease commences in a slow manner, the patient not even complaining of chilliness, it may be called subacute, and must be treated more mildly. If there be no tenderness of the epigastrium, and if the brain be not affected, the treatment may be commenced with an emetic, after which a dose of calomel and jalap or rhubarb should be administered, and repeated every few hours until it operates. So soon as the bowels are cleared, antimonials should be given and continued as directed above. Five grains of calomel, or more, should be given every four or six hours until the mouth is made slightly sore, or the general system is affected, which will be known by an increased fulness and regularity of the pulse. When this is brought about, all has been done that can be advantageously accomplished by mercury in large doses. As in acute cases the action of the liver and the secretions and excretions of the abdominal viscera, generally, should be kept in a proper state of activity. This can be best done by calomel or the blue pill. I am much in favor of the latter in female patients, and in all delicate or enfeebled constitutions. One or two alvine evacuations should be procured daily through the fore and middle part of the fever; but if it continue for a long time, as for more than two weeks, I would only wish one evacuation a day, or one every other, or every third day, if the patient should be very low. Rhubarb, combined with compound extract of colocynth and a small proportion of ipecacuanha, will form a very good combination for the purpose; the physician will, however, find jalap, salts, and senna, and magnesia to answer a good purpose.

When there is local inflammation or congestion of the brain, or of any other part of the system, whether in the acute or subacute form of the disease, attention must be given to it. If in the brain or its membranes, keeping the hair wet with cold or tepid water, will be of use. Cupping on the temples, or back of the neck, will also be of much service. Should these not relieve, I would advise blistering on the head or neck. When there is pain in the small of the back, cupping and blistering will be of much service. If the stomach be irritable, or if there be tenderness of the epigastrium, cupping should be used and after it blistering. If the patient be weak, it would be better not to scarify. If there be pain or tenderness generally diffused over the abdomen, cupping should never be neglected. Half a dozen cups either with or without scarification, will give great relief, and this may be repeated several times, if the irritability continue. Local applications may be used with advantage.

It is a matter of moment in the treatment of this fever, that nothing shall be done to bring the pulse below the natural standard for any length of time. I would always prefer having a pulse a little above this standard. When it falls below means should be taken to raise it. This is effected by the addition of camphor, ammonia, good wine or brandy. Opium will often be found useful, particularly when combined with ipecacuanha, but I think five grain doses of camphor with a grain of ipecacuanha, if the latter be admissible will answer a better purpose than most other stimulants. Sulphate of morphia or opium may be added if thought proper. If these three medicines be given in combination, when there is a disposition to sweat, their effects will be most salutary. I should not neglect to observe that sulphate of quinine if given in moderate doses, is a medicine of great value in the treatment of this fever when much debility is present.

Much has been said for and against bleeding in the cure of this fever, and it has been urged that physicians who have been in the habit of bleeding for it have often been unfortunate; this I cannot deny. Bleeding is but seldom necessary as the warm or cold affusion will generally moderate the force of the fever more safely than bleeding; indeed I would advise the young practitioner to resort to the lancet with much caution, for if he bleeds so that the pulse does not regain a firm beat he will assuredly lose his patient.

Sometimes after this fever has existed for a number of days, or even weeks, hemorrhage from the bowels takes place. This may be feared, when the tongue of a sudden loses a thick coat, which comes off in flakes, and leaves the surface smooth and red with a polished appearance, and in cases where but little emaciation has taken place, and where there is fulness of the abdomen. The most common cause of its appearance is excessive purgation for a number of days. This is particularly the case where calomel has been given in large quantities at the same time that cold water has been administered largely. Some practitioners never give up the administration of calomel in frightful doses, until their patients are either salivated or poisoned to death. Uninterrupted purging is wrong, and giving mercury with an idea that it does no good unless the patient be salivated is as bad. This most potent medicine is shamefully abused in this way, and I am not certain but that its indiscriminate exhibition with cold drinks often has a most injurious tendency. When hemorrhage occurs it becomes necessary at once to cease giving the purgative medicine, and to give opiates every six or eight hours, until the bleeding ceases. The addition of ipecacuanha to the opium will be useful, and if but little mercury has been administered, moderate quantities of calomel should be combined with the opium or morphine. Dry cupping will be important over the abdomen, after which a blister should be applied. Purging must not be attempted under four or five days, when rhubarb or castor oil should be given slowly until the operation is procured; when this is effected, great care should be taken to avoid frequent purging. Quinine is often of much use after great loss of blood in this way; wine and brandy are also useful. The first cases of this affection which I saw all terminated unfavorably; indeed I at one time almost came to the conclusion that death was inevitable, but eventually being called to a case in my own practice, where the family had given large doses of salts, I concluded that I would try calomel, opium and ipecacuanha, and I had the satisfaction of saving my patient, and have not lost one since with bleeding from the bowels. I did not suffer the patient in this case to take any cathartic medicine for more than a week. However, in about five days he had an operation from the effects of the calomel. When I first saw this patient I thought all was over with him as I found him in a fainting condition from the loss of blood. Since the time I attended this case I have seen several nearly as bad and some much worse, yet they all recovered.

In conclusion I may observe, that if the method which I have laid down for the treatment of this fever be pursued but few cases will be lost. During a residence as a practitioner of medicine in this district of country, which I have described, for eighteen years, I lost but very few cases, probably not more than one in each year, and indeed I shall always look back with pleasure upon my success in the treatment of these cases.

Oct., 1841.

